# Impact of Betamethasone Pretreatment on Engrafment of Cord Blood-Derived Hematopoietic Stem Cells

**DOI:** 10.1007/s00005-022-00666-5

**Published:** 2022-12-18

**Authors:** David Perna-Barrull, Laia Gomez-Muñoz, Silvia Rodriguez-Fernandez, Anna Gieras, Rosa M. Ampudia-Carrasco, Lidia Almenara-Fuentes, Ruth M. Risueño, Sergi Querol, Eva Tolosa, Marta Vives-Pi

**Affiliations:** 1 Immunology Department, Germans Trias I Pujol Research Institute, Autonomous University of Barcelona, Badalona, Spain; 2 Department of Immunology, University Medical Center Hamburg-Eppendorf, Hamburg, Germany; 3 Ahead Therapeutics SL, Barcelona, Spain; 4 Josep Carreras Leukaemia Research Institute, Campus IGTP-ICO, Badalona, Spain; 5 Cell Therapy Services and Cord Blood Bank, Catalan Blood and Tissue Bank, Barcelona, Spain

**Keywords:** Betamethasone, Cord blood, Hematopoietic stem cell, CD34^+^, Transplantation, Immune system

## Abstract

Hematopoietic stem cell (HSC) transplantation is crucial to cure hematologic malignancies. Umbilical cord blood (UCB) is a source of stem cells, but 90% of UCB units are discarded due to low cellularity. Improving the engraftment capacities of CD34^+^ stem cells would allow the use of UCB that were so far rejected. Betamethasone induces long-term transcriptomic and epigenomic changes in immune cells through glucocorticoid receptor. We hypothesize that discarded UCB could be used owing to improvements induced by betamethasone. Isolated CD34^+^ HSC from UCB were exposed to the synthetic glucocorticoids betamethasone and fluticasone for 20 h, and cell phenotype was determined before transplantation. NSG mice were sub-lethally irradiated (1 Gy or 2 Gy) 6 h before intravenously transferring 2–5 × 10^5^ CD34^+^ HSC. The peripheral blood engraftment levels and the leukocyte subsets were followed up for 20 weeks using flow cytometry. At end point, the engraftment and leukocyte subsets were determined in the spleen and bone marrow. We demonstrated that betamethasone has surprising effects in recovering immune system homeostasis. Betamethasone and fluticasone increase CXCR4 and decrease HLA class II and CD54 expression in CD34^+^ HSCs. Both glucocorticoids-exposed cells showed a similar engraftment in 2 Gy-irradiated NSG mice. Interestingly, betamethasone-exposed cells showed enhanced engraftment in 1 Gy-irradiated NSG mice, with a trend to increase regulatory T cell percentage when compared to control. Betamethasone induces alterations in CD34^+^ HSCs and improve the engraftment, leading to a faster immune system recovery, which will contribute to engrafted cells survival.

## Introduction

Synthetic glucocorticoids (e.g., prednisolone, fluticasone, or betamethasone) are drugs widely used for their immunomodulatory effects. Their mechanism of action is through the interaction with transcription factors related to inflammation, such as NF-κB and AP-1 (Cain and Cidlowski 2017), inhibiting their function. These molecules also induce anti-inflammatory pathways such as interleukin (IL)-10 production (Mozo et al. 2004). Short-term exposure to synthetic glucocorticoids induces long-term changes in gene expression of immune system cells (Franco et al. 2019; Hong et al. 2020; Wang et al. 2019). Moreover, glucocorticoids can exert a rapid, non-genomic effect on cells through kinases and the release of accessory proteins (Ramamoorthy and Cidlowski 2016). Betamethasone—usually administered to pregnant women at risk of pre-term delivery—induces fetal lung maturation, thus reducing mortality and morbidity in pre-term newborns (Liggins and Howie 1972). Betamethasone targets the ubiquitous glucocorticoid receptor, present in almost all cell types, including stem cells and leukocytes, and may therefore affect the development of the immune system (Solano et al. 2016).

We previously demonstrated that betamethasone increases self-tolerance and arrests autoimmunity in experimental type 1 diabetes (T1D) by inducing changes in leukocytes and islet β cells (Gieras et al. 2017; Perna-Barrull et al. 2019). In this sense, betamethasone alters gene expression and causes long-term changes through histone acetylation and DNA methylation (Kim et al. 2022; Seckl 2004). These alterations modify the accessibility to enhancers that control the expression of relevant molecules. Other synthetic glucocorticoids, such as fluticasone propionate—usually administered to patients with asthma—induce similar effects.

Hematopoietic stem cell transplantation (HSCT) refers to the transplantation of stem cells for the treatment of hematologic, autoimmune, and genetic diseases. Malignant hematologic diseases have an incidence of 40/100,000 inhabitants per year in Europe and are particularly dramatic in children, who show a survival rate of 45–50% five years after HSCT (Copelan 2009; Styczyński et al. 2020). The main source of stem cells for allogeneic transplantation is bone marrow or mobilized peripheral blood from adult donors, but the procedure is limited since it is restricted by the human leukocyte antigen (HLA) matching. Umbilical cord blood (UCB) is an alternative source of stem cells for allogeneic transplantation with clear benefits, including a lower HLA match requirement and the ease of retrieval in a non-invasive manner. Engraftment success of UCB correlates with the number of stem cells (CD34^+^) transplanted, being the reason why only 10% of the cord blood donations are used for HSCT (Rebulla et al. 2022; Wagner et al. 2002).

Here, we provide a strategy to improve the engraftment capacities of UCB stem cells using betamethasone as a pre-conditioning treatment. We show that cord blood CD34^+^ hematopoietic stem cells (HSC) exposed to betamethasone increase the expression of C-X-C chemokine receptor type 4 (CXCR4) and decrease the expression of HLA class II and CD54 without compromising their viability and function. These changes correlate with an improvement of CD34^+^ cell engraftment in suboptimal (low-irradiation) conditions.

## Materials and Methods

### Mice

Immunodeficient NOD.SCID-IL2Rɣ^−/−^ (NSG) mice were obtained from Charles River Laboratories (Barcelona, Spain) and maintained in the Animal Care Facility of Germans Trias i Pujol Research Institute. NSG mice were kept under specific pathogen-free conditions, in a 12 h dark/12 h light cycle with food and water ad libitum. Mice were anesthetized using Isoflurane (Forane, Abbvie pharmaceutics, Madrid, Spain) and euthanized by cervical dislocation. All animal studies were approved by the institutional animal ethics committee.

### Cell Isolation

UCB samples were obtained from the Blood and Tissue Research Biobank (Barcelona, Spain). All samples were obtained between 20 and 72 h after delivery. To isolate CD34^+^ cells, UCB were incubated with RosetteSep (STEMCELL Technologies, Vancouver, Canada) for 20 min prior to density gradient purification using Ficoll-Paque (GE Healthcare, Uppsala, Sweden) and centrifugation at 700*g* for 30 min. CD34^+^ cells were selected from the mononuclear cells fraction using the EasySep human cord blood CD34^+^ selection kit II (STEMCELL Technologies, Vancouver, Canada). Briefly, mononuclear cells were incubated with anti-human CD34 antibodies and FcR-blocking antibodies for 10 min. Then, dextran microbeads were added to the cell suspension and incubated for 1 min at room temperature. The immunomagnetic selection was performed using EasySep Magnets (STEMCELL Technologies) according to the manufacturer’s instructions. Purity, viability, and cell number were then assessed by flow cytometry using anti-human CD34-PE (BD Biosciences, San Jose, CA, USA), anti-human CD45-FITC (BD Biosciences), 7-amino-actinomycin-D (7-AAD, BD Biosciences) and Perfect Count Microbeads (Cytognos, Salamanca, Spain). Only samples with cell purity higher than 75% and viability higher than 90% were used.

### Cell Culture

CD34^+^ stem cells were cultured at a density of 1 × 10^5^ CD34^+^/mL with RPMI-1640 (Biowest, Nuaille, France) complete medium (CM) containing 10% fetal bovine serum (Gibco, Invitrogen, Carlsbad, CA, USA), penicillin (100 U/ml, Normon SA, Madrid, Spain), streptomycin (100 ug/mL, Reig Jofre, Sant Joan Despí, Spain), glutamine (2 mmol/mL, Sigma-Aldrich, St. Louis, MO, USA), sodium pyruvate (1 mmol/L, Gibco) and beta-mercaptoethanol (1 mmol/L, Sigma-Aldrich). Moreover, CM was supplemented with the following human growth factors: Stem cell factor (100 ng/mL), FMS-related tyrosine kinase 3 ligand (100 ng/mL), IL-6 (50 ng/mL) and thrombopoietin (40 ng/mL; Preprotech, London UK). Depending on the group condition, cells were cultured with betamethasone (100 nM, Sigma-Aldrich), fluticasone propionate (100 nM, Fluticasone, Selleckchem, Houston, TX, USA), or phosphate-buffered saline (PBS). Glucocorticoid concentration was chosen based on our previous results (Perna-Barrull et al. 2019). Cells were incubated for 20 h at 37 ºC in a humidified atmosphere containing 5% CO_2_. Before transplantation, viability and CD34^+^ purity were assessed by flow cytometry using anti-human CD34-PE (BD Biosciences), anti-human CD45-FITC (BD Biosciences), and 7-AAD (BD Biosciences). In addition, cell number was determined using Perfect Count Microbeads (Cytognos, Salamanca, Spain).

### Pre-Engraftment Phenotype of CD34^+^ Cells

The phenotype of CD34^+^ HSC exposed to betamethasone or fluticasone after 20 h of culture was determined by flow cytometry. To that end, 20.000 CD34^+^ cells of each group (control, betamethasone, and fluticasone) were stained with anti-human CD34 PE (BD Biosciences), HLA-ABC FITC (Immunotools), CD45 PercP (BD Biosciences), CD40 APC (Immunotools), CD54 PE-Cy7, CXCR4 APC-Cy7 and HLA-DR V500 (BD Biosciences), and median fluorescence intensity values were obtained using FACS Canto II cytometer (BD Biosciences). Data were analyzed using FlowJo software (Tree Star Inc., Ashland, OR, USA).

### CD34^+^ Cord Blood Cell Transplantation

Mice were transplanted at 8–10 weeks of age. Before transplantation, mice were sub-lethally whole-body irradiated at 1 or 2 Gy using a cesium source (Scherin, IBL 437 C). The standard irradiation in NSG is from 2 to 4 Gy (McDermott et al. 2010). We considered 2 Gy as optimal irradiation and 1 Gy as suboptimal irradiation. After irradiation, mice were randomized into treatment groups. Six hours after irradiation, mice transplantation was performed by transferring 2–5 × 10^5^ CD34^+^ cells obtained after 20 h of cell culture exposed to betamethasone (100 nM, Sigma-Aldrich) or fluticasone (100 nM, Selleckchem). As a control, the same dose of non-glucocorticoid exposed CD34^+^ cells from the same UCB was used. Cells were injected intravenously into the tail vein. For each experimental group, 3–5 mice were transplanted.

### Flow Cytometry

To assess changes induced by betamethasone in leukocyte subsets of transplanted mice, peripheral blood was obtained every 1–2 weeks for 20 weeks. Moreover, bone marrow and spleens were harvested at the endpoint of the study, and single-cell suspensions were obtained by mechanical disruption. Cells were stained with anti-human CD3 BV650, CD4 AF488, CD8a AF700, CD1c PE, CD14 BV711, CD16 BV605, CD20 AF700, CD25 BV421, CD45 BV510, CD56 APC, CD123 PE-Cy7, CD127 PerCP Cy5.5, HLA-DR BV785 (all from Biolegend, San Diego, CA, USA), CD11c PE-efluor610 (eBioscience, San Diego, CA, USA), TCR gd PE-Cy7 (BD Biosciences), anti-mouse CD45 AF700 (eBioscience) and Alexa Fluor 750 (Thermo Fisher Scientific, Waltham, MA, EUA) for viability. Peripheral blood cells were directly stained, and RBC lysis/Fixation solution (Biolegend) was used before acquisition. The acquisition was performed using LSR Fortessa cytometer (BD Biosciences). Data were analyzed using FlowJo software (Tree Star Inc). Chimerism percentages were calculated as human leukocyte counts (HuCD45^+^) with respect to the total leukocyte counts (HuCD45^+^ and muCD45^+^). Human leukocyte subsets were defined as specified in Table 1.

**Table 1:** List of markers of different leukocyte subsets analyzed

Leukocyte subset	Phenotype
Monocytes	CD45^+^ CD20^−^ CD3^−^ HLA-DR^+^ CD14^+^
Conventional dendritic cells CD1c^+^ (cDC2)	CD45^+^ CD20^−^ CD3^−^ HLA-DR^+^ CD123^−^ CD14^−^ CD11c^+^ CD1c^+^
Plasmacytoid dendritic cells	CD45^+^ CD20^−^ CD3^−^ HLA-DR^+^ CD123^hi^
B cells	CD45^+^ CD20^+^ CD3^−^
T cells αβ	CD45^+^ CD20^−^ CD3^+^ CD56^−^ TCRγδ^−^
CD4^+^ T cells	CD45^+^ CD20^−^ CD3^+^ CD56^−^ TCRγδ^−^ CD4^+^ CD8^–^
CD8^+^ T cells	CD45^+^ CD20^−^ CD3^+^ CD56^−^ TCRγδ^−^ CD4^–^ CD8^+^
Regulatory T cells	CD45^+^ CD20^−^ CD3^+^ CD56^−^ TCRγδ^−^ CD4^+^ CD8^−^CD127^−^ CD25^+^

### Statistical Analysis

Statistical analysis was performed using Prism 9.3 software (GraphPad Software Inc., San Diego, CA, USA). For comparison between groups, a Friedman’s test with a posthoc Dunn’s test or Mixed-effect analysis with Geisser–Greenhouse correction and a post hoc Tukey’s multiple comparison test were used. For comparisons between treatments in the follow-up experiments, a Wilcoxon test for the area under the curve (AUC) was used, and differences between weeks in the follow-up experiment for each group were assessed with a Tukey’s (three treatments) or Wilcoxon (two treatments) multiple comparisons test. For paired data, a non-parametric Wilcoxon test was used.

## Results

### Betamethasone Alters the Phenotype of CD34^+^ Cord Blood Cells

**Fig. 1:**
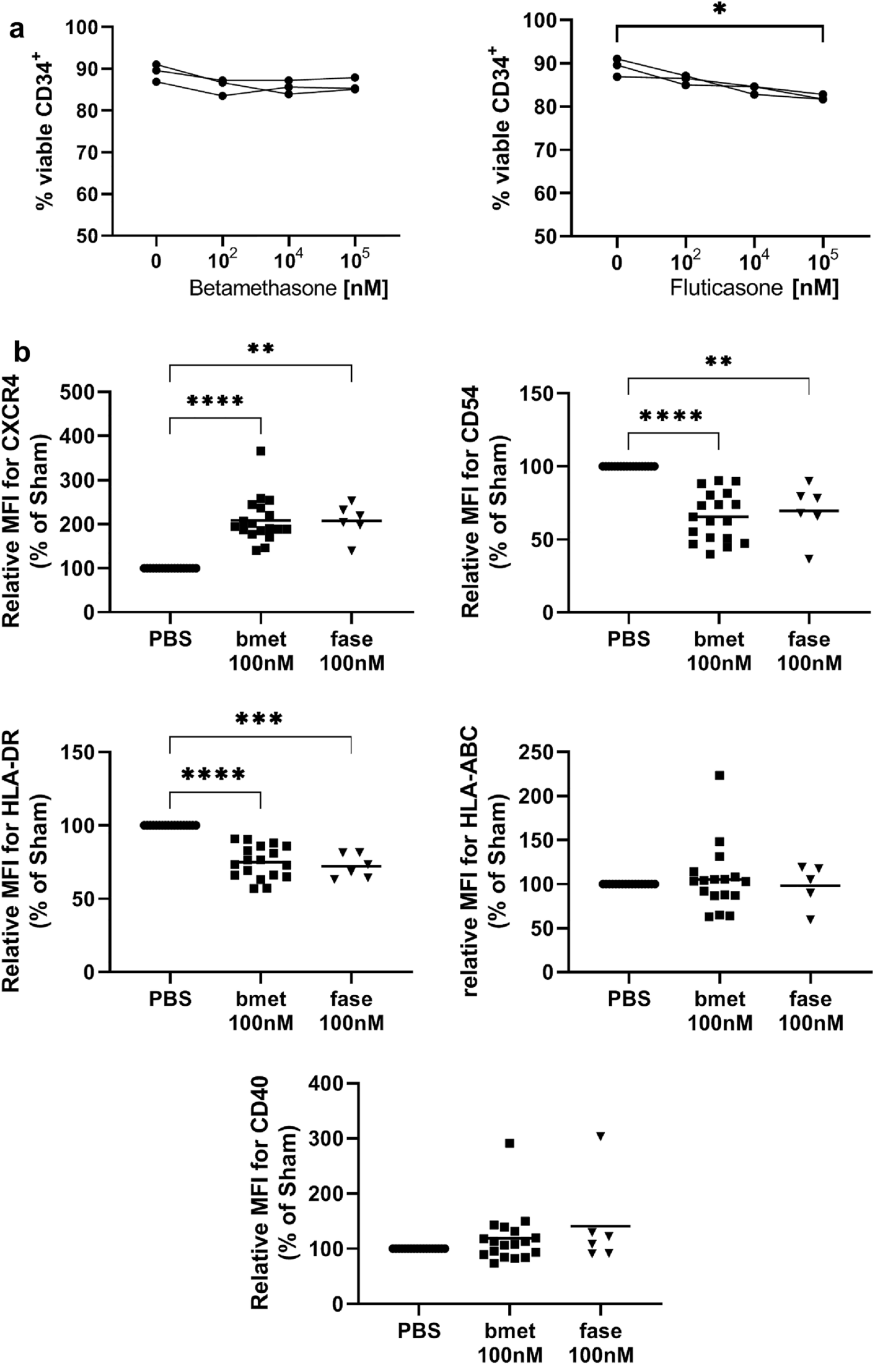
Effect of betamethasone on CD34^+^ phenotype. (**a**) Percentage of viable CD34^+^ cells (annexin-V PE^–^, 7aad^–^) after 20 h cultured with betamethasone (*left panel*) and fluticasone (*right panel*) at 10^2^, 10^4^ and 10^5^ nM. Dots show three independent experiments and lines connect paired experiments (*p ≤ 0.05, Dunn’s test, Friedman test). (**b**) Relative median fluorescence intensity (MFI) of CXCR4, CD54, MHC class II (HLA-DR), MHC class I, and CD40 expression on the membrane of CD34^+^ cells. PBS represents control conditions. Squares and triangles represent CD34^+^ cells cultured with 100 nM betamethasone (bmet) or 100 nM fluticasone (fase) respectively. Lines show the mean of *n* = 6–18 independent experiments. (*p ≤ 0.05, **p < 0.01, ***p < 0.001, ****p < 0.0001, Mixed-effect analysis with the Geisser–Greenhouse correction, Tukey’s multiple comparison test)

The direct effect of betamethasone on CD34^+^ cord blood cells was tested in vitro. The viability of CD34^+^ cells was not impaired after 20 h in culture with betamethasone at 10^2^, 10^4^ or 10^5^ nM (Fig. 1a). By contrast, fluticasone, another synthetic glucocorticoid with described effect on CD34^+^ cord blood cells, significantly decreased the viability of CD34^+^ cells at 10^5^ nM (*p* < 0.05). Then, phenotypic changes induced by 20 h exposure to betamethasone and fluticasone were assessed. Figure 1b shows a significant increase in CXCR4, and a significant reduction in CD54 and HLA-DR by betamethasone (*p* < 0.0001) and fluticasone (*p* < 0.01, 0.05 and 0.001, respectively) on CD34^+^ cells in comparison to the control group. No differences were found in HLA class I and CD40 expression. In summary, betamethasone alters CD34^+^ cells toward a less immunogenic phenotype and a potentially improved repopulation.

### Betamethasone Allows Experimental CD34^+^ Cell Engraftment

**Fig. 2:**
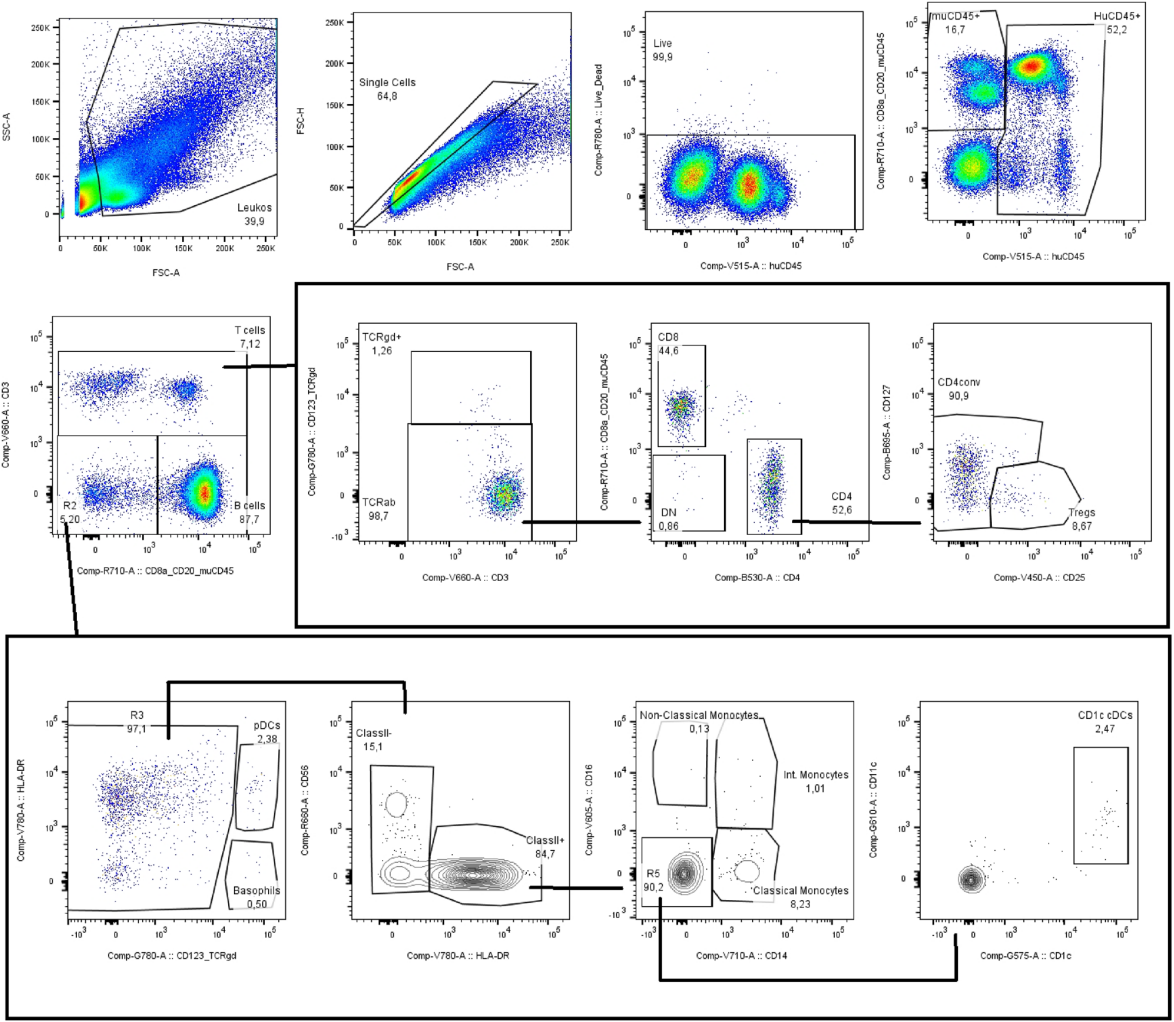
Representative gating strategy for the analysis of the engraftment and leukocyte subsets. The gating strategy was used during the follow-up in peripheral blood, and at the end of the study in the spleen and bone marrow to determine the engraftment and leukocyte subsets as defined in Table 1

**Fig. 3:**
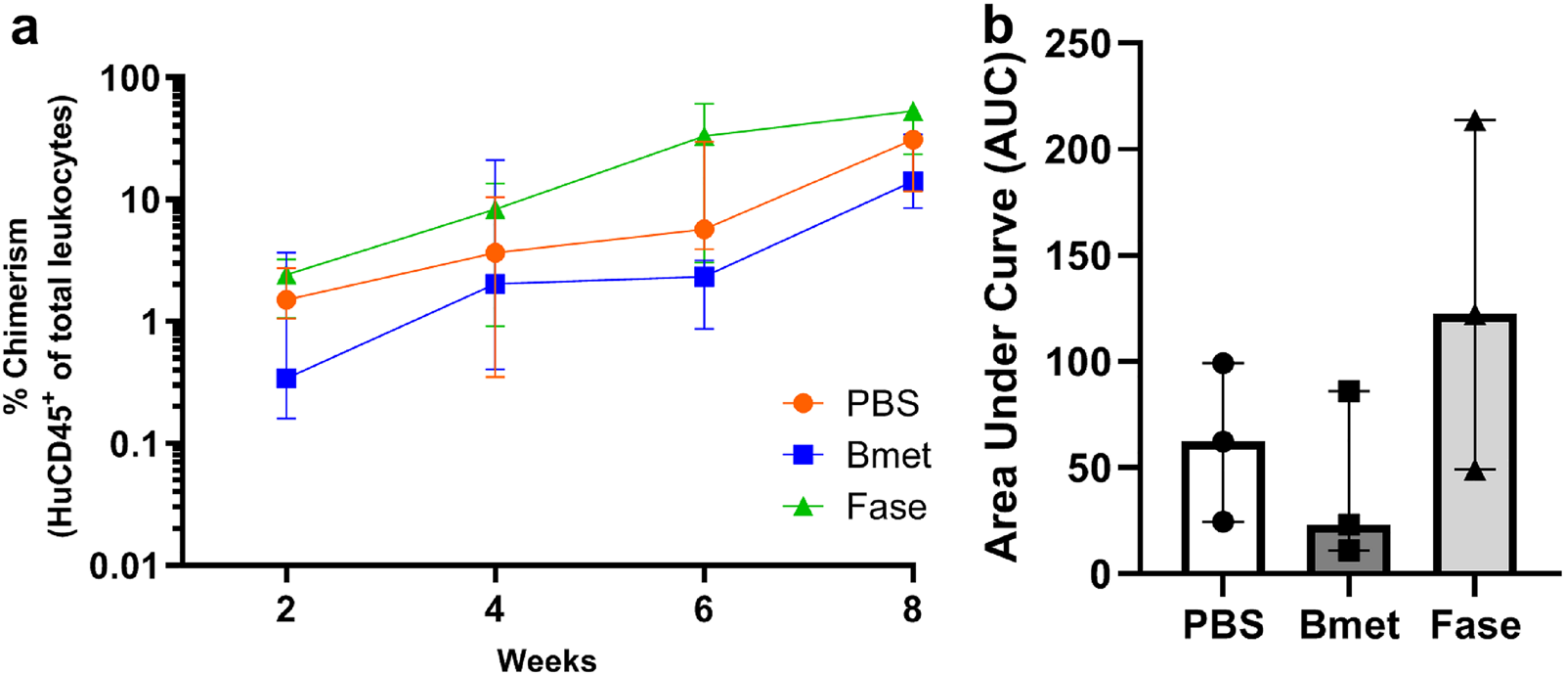
Repopulation kinetics in mice irradiated with 2 Gy (optimal conditions). Human chimerism was determined at 2, 4, 6, and 8 weeks post transplantation in peripheral blood (three animals per group). **(a)** The percentage of chimerism in peripheral blood of mice transplanted with HuCD34^+^ cultured for 20 h with PBS (orange dot and line show the median and interquartile range, IQR), betamethasone (Bmet, blue square and line show the median, IQR) or fluticasone (Fase, green triangle and line show the median, IQR) after 8 weeks. At the end of the follow-up, the median (IQR) percentage of chimerism reached 30.91 (11.65–31.71), 14.10 (8.52–34.03) and 53.16 (23.41–62.35), respectively (Tukey’s multiple comparison test). **(b)** Area under the curve (AUC) at week 8 post transplantation, bars show the median (IQR) of three mice (Dunn’s test, Friedman test)

To assess the effect of betamethasone on CD34^+^ HSC engraftment in optimal conditions, mice were irradiated at 2 Gy, and cord blood CD34^+^ HSC were cultured with betamethasone or fluticasone at 100 nM for 20 h before transplantation. Fluticasone-treated cells were used as a positive control because it has been described that this drug improves cell engraftment (Guo et al. 2017). Before transplantation, the purity of the HuCD34^+^fraction was 93.02% ± 2.80% (mean ± SD) and viability was always > 90% in the three conditions (PBS, Bmet, and Fase). In the present study, the gating strategy (Fig. 2) was used to determine the engraftment and leukocyte subsets, as defined in Table 1. No significant differences were observed in the engraftment between the control (PBS), betamethasone, and fluticasone groups (Fig. 3a). The percentage of human chimerism at 2, 4, 6, and 8 weeks after transplantation was similar in all conditions. These results demonstrate that betamethasone-treated cells do not impair CD34^+^ HSC engraftment in this experimental model. The analysis of the AUC confirmed these results (Fig. 3b).

### Betamethasone-Treated CD34^+^ Cells Improve Engraftment in Suboptimal Conditions

**Fig. 4:**
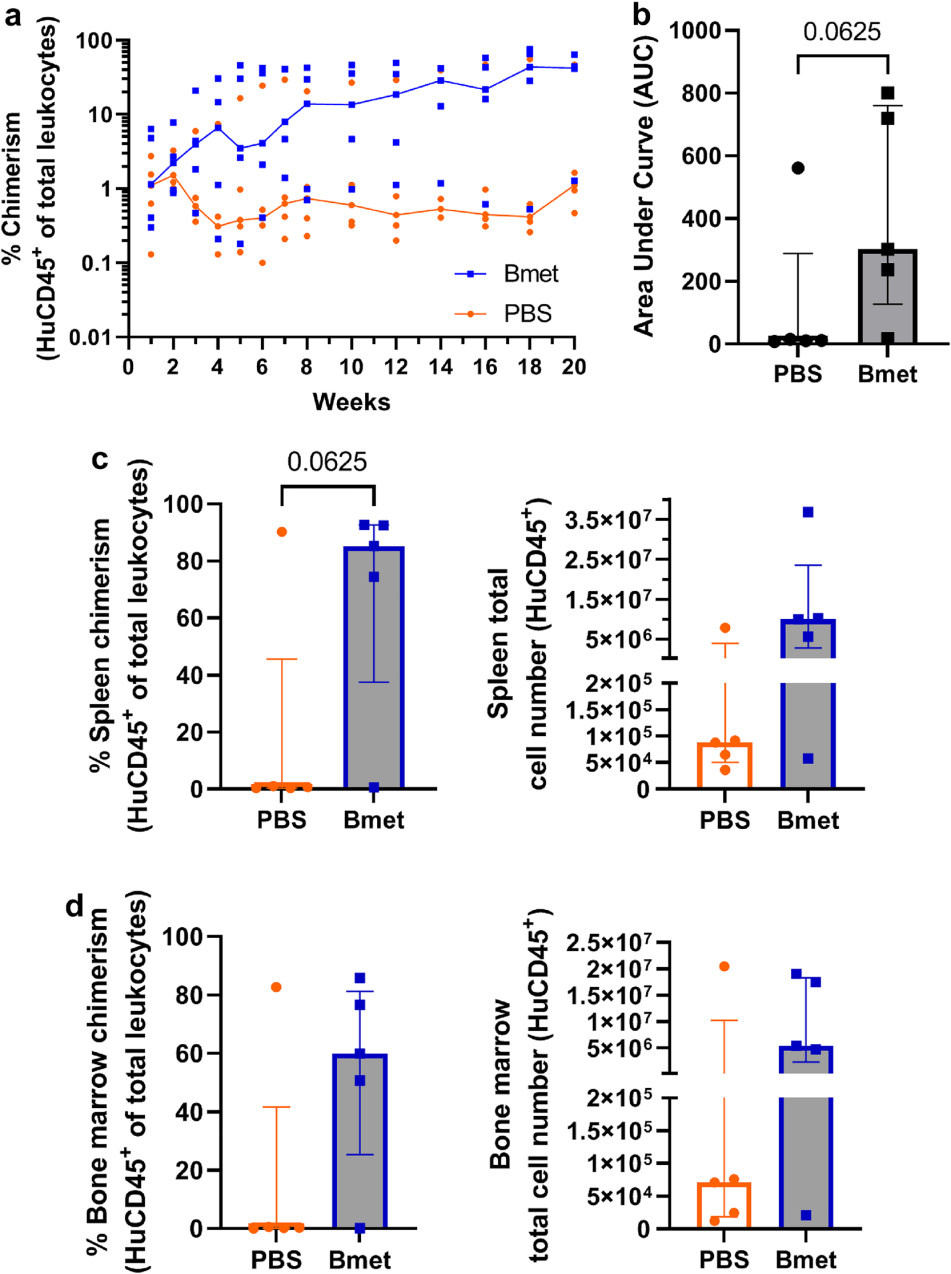
Repopulation kinetics in mice irradiated with 1 Gy (suboptimal conditions). Human chimerism was determined at 1–20 weeks post transplantation in peripheral blood. At week 20, chimerism was determined in the spleen and bone marrow (five animals per group). **(a)** The repopulation of 20 h unexposed (PBS) or betamethasone-exposed (Bmet) CD34^+^ cells at week 20 post transplantation reached a median (interquartile range, IQR) percentage of chimerism of 1.12 (0.71–24.05) and 42.29 (21.21–54.40), respectively. Lines show the median percentage of chimerism in 5 mice (Wilcoxon multiple comparisons test). **(b)** Area under the curve (AUC) at week 20 post transplantation chimerism in peripheral blood of betamethasone-exposed (Bmet) or unexposed (PBS) HuCD34^+^ cells, bars show the median (IQR) of five mice (Wilcoxon matched-paired test). **(c)** Percentage (left panel) and total cell number (right panel) of betamethasone-exposed (blue squares) or unexposed (orange dots) HuCD34^+^ cells in the spleen of mice 20 weeks after transplantation or **(d)** in the bone marrow. Bars show the median (IQR) percentage of leukocyte subset chimerism of five mice (Wilcoxon matched-paired test)

Because myeloablative irradiation has hematopoietic, gastrointestinal, and neurological side effects, we evaluated the ability of betamethasone-treated CD34^+^ cells to engraft in suboptimal conditions of irradiation (sublethal low irradiation, 1 Gy). Using 1 Gy the niche space in the bone marrow will be limited, and HSC will compete with other cells. Before transplantation, both betamethasone-exposed and unexposed HuCD34^+^ cells showed viability higher than 90%. Figure 4a shows peripheral blood chimerism over a period of 20 weeks post transplantation. We considered successful engraftment when more than 2% of HuCD45^+^ of total leukocytes were detected 10 weeks after transplantation. In the control group, only one mouse showed positive engraftment, whereas 4/5 mice transplanted with betamethasone-exposed CD34^+^ cells displayed positive engraftment. Thus, a clear trend to improve the chimerism was observed at weeks 7, 12, 14, and 20 when cells were treated with betamethasone before transplantation (*p* = 0.0625). The AUC (Fig. 4b) confirmed this biological effect, although the difference was not significant (*p* = 0.0625). At the end of the study (20 weeks post transplantation), chimerism was also assessed in the spleen and bone marrow. The percentage and number of HuCD45^+^ cells with respect to total leukocytes tended to be higher in the spleen (Fig. 4c, *p* = 0.0625) and bone marrow (Fig. 4d) of mice transplanted with CD34^+^ exposed to betamethasone.

### Follow-up of the Development of Human Immune Cell Subsets after Transplant with Human CD34^+^ Exposed to Betamethasone

**Fig. 5:**
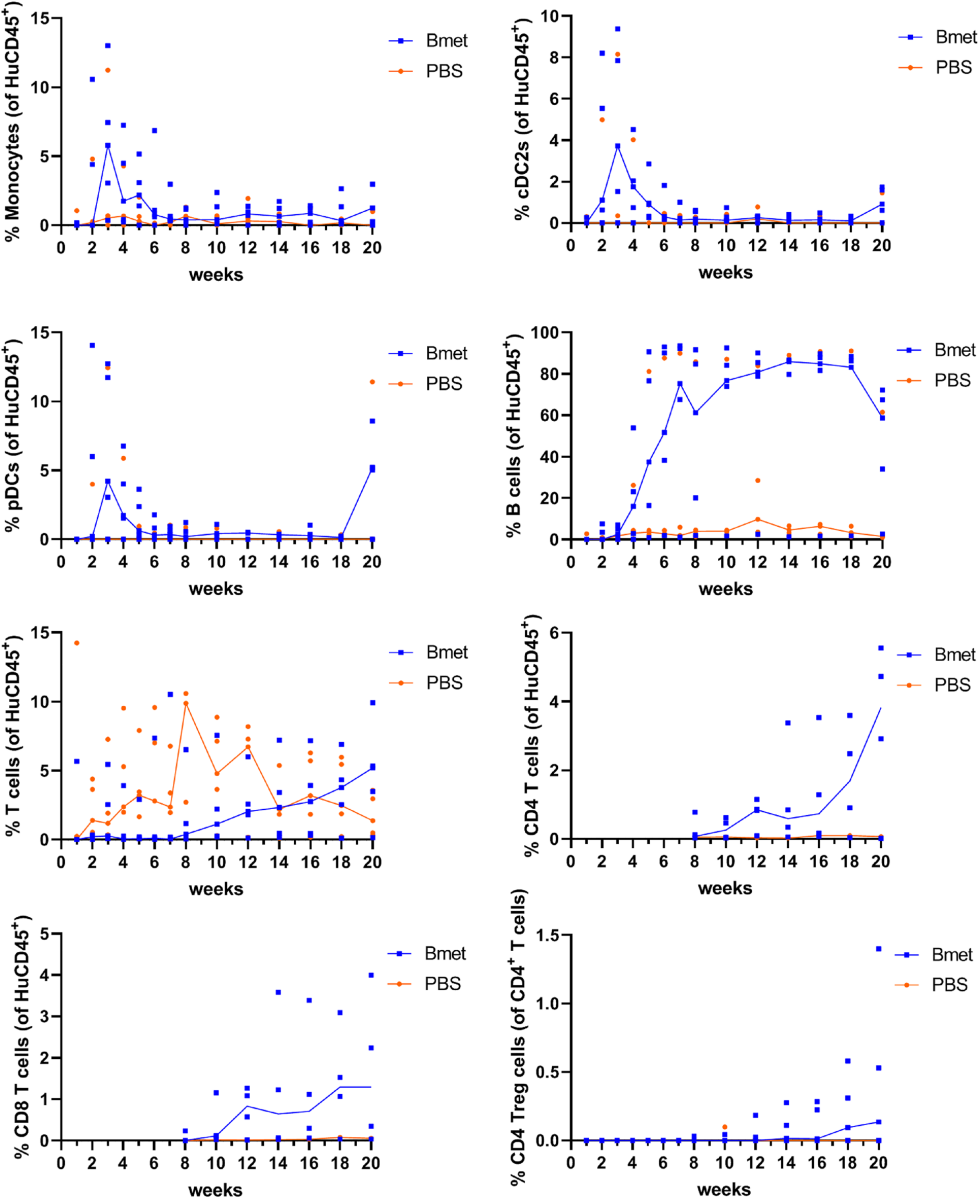
Engraftment of human immune cell subsets in peripheral blood samples. Percentage of human subsets assessed by flow cytometry at 1–20 weeks after transplantation in mice (five animals per group). The analyzed subsets (as defined in Table 1) were monocytes, conventional dendritic cells CD1c^+^ (cDC2s), plasmacytoid dendritic cells (pDCs), B lymphocytes, T lymphocytes, CD4^+^ T cells, CD8^+^ T cells, and CD4^+^ Treg cells from mice transplanted with betamethasone-exposed HuCD34^+^ cells (Bmet, blue squares, the blue line shows the median percentage of subsets in five mice) or unexposed HuCD34^+^ cells (PBS, orange dots, the orange line shows the median percentage of subsets in five mice) (Wilcoxon multiple comparisons test)

We next aimed at assessing the quality of immune cell reconstitution by determining the percentages of different human immune cell subsets in peripheral blood of low-irradiated transplanted mice during the study follow-up. Table 1 shows the phenotype of the different leukocyte subsets analyzed. Figure 5 shows the evolution of different cell subsets in NSG mice transplanted with CD34^+^ cord blood cells exposed to betamethasone in comparison to unexposed cells. All subsets showed improved biological expansion after betamethasone exposure. The percentage of monocytes, conventional dendritic cells CD1c^+^ (cDC2s), and plasmacytoid dendritic cells (pDCs) showed a trend to increase 2–4 weeks after transplant, decreasing then from week 6 in both groups. By contrast, B cells were detected 4 weeks after transplant reaching a plateau at weeks 7–9. At that point, the median (interquartile range) B cell percentage (of HuCD45^+^ cells) was 75.26 (34.36–92.86) in the betamethasone group and 1.95 (1.43–47.93) in the control group. As expected, T cells arise 8 weeks after transplantation, both CD4^+^ and CD8^+^, increasing in percentage until the end of the follow-up and always to a greater extent in the betamethasone group. CD4^+^ regulatory T cells (Treg) were detected only in the betamethasone group from 12 to 14 weeks after transplant.

### Spleen and Bone Marrow Repopulation Increases with Betamethasone-Exposed CD34^+^ Cord Blood Cells

**Fig. 6:**
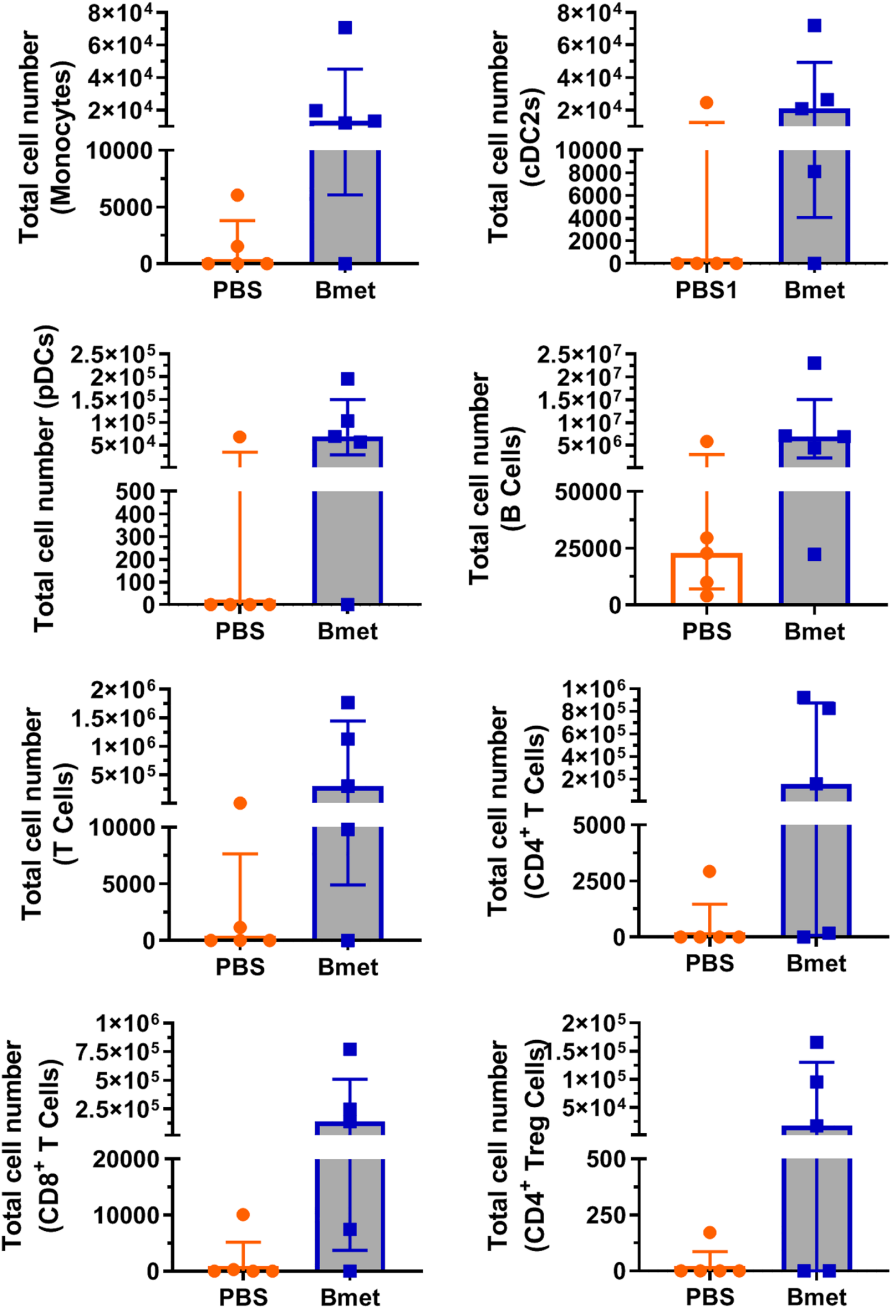
Total cell number of human immune cell subsets in the spleen. The total cell number of human subsets was assessed by flow cytometry in the spleen of mice at week 20 after transplantation. Bars show the median (interquartile range) number of subset cells in five animals per group. The analyzed subsets (as defined in Table 1) were monocytes, conventional dendritic cells CD1c^+^ (cDC2s), plasmacytoid dendritic cells (pDCs), B lymphocytes (B cells), T lymphocytes (T cells), CD4^+^ T cells, CD8^+^ T cells, and CD4^+^ Treg cells (Wilcoxon matched-paired test)

**Fig. 7:**
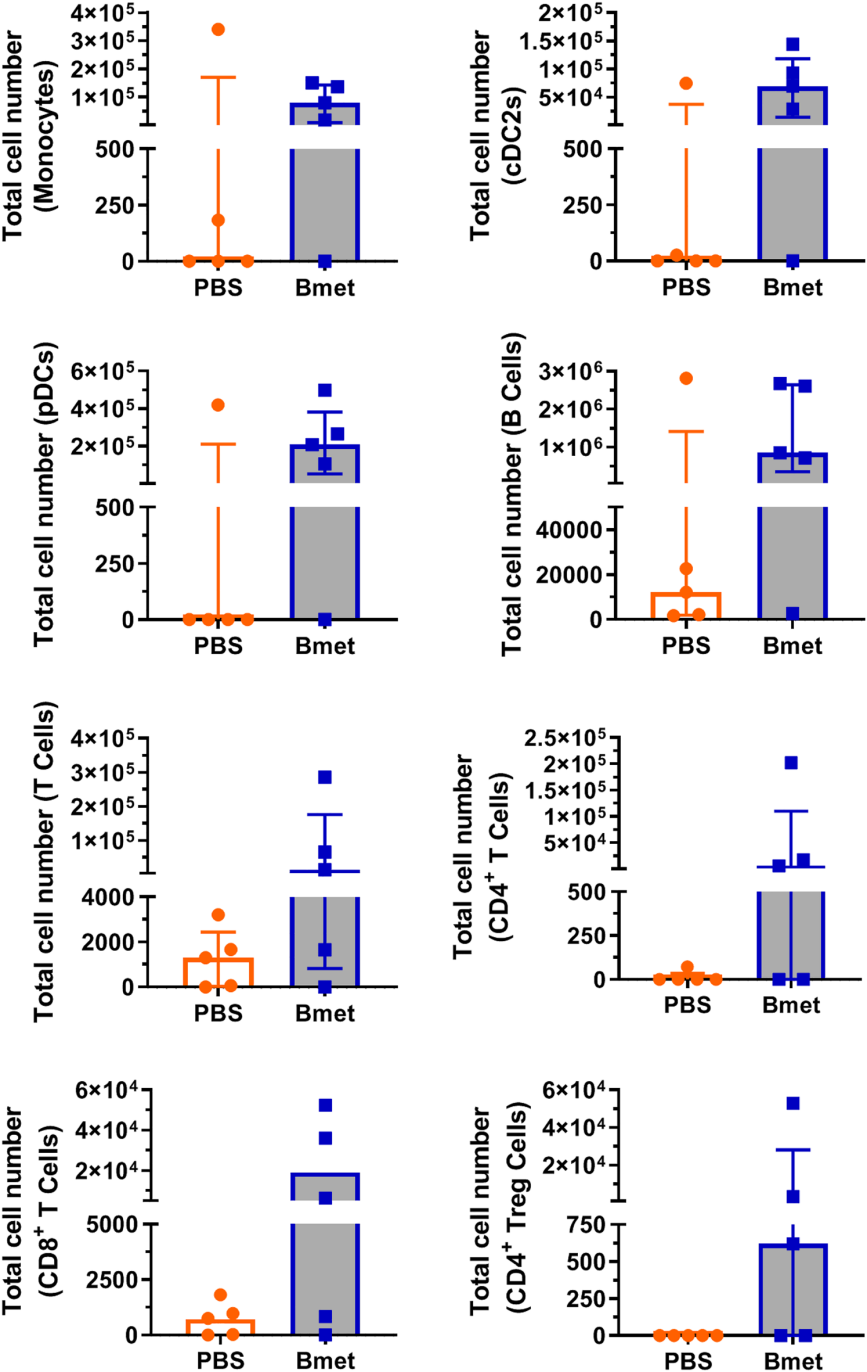
Total cell number of human immune cell subsets in the bone marrow. The total cell number of human subsets was assessed by flow cytometry in the bone marrow of mice at week 20 after transplantation (bars show the median (interquartile range) of five animals per group). The analyzed subsets (as defined in Table 1) were monocytes, conventional dendritic cells CD1c^+^ (cDC2s), plasmacytoid dendritic cells (pDCs), B lymphocytes (B cells), T lymphocytes (T cells), CD4^+^ T cells, CD8^+^ T cells, and CD4^+^ Treg cells (Wilcoxon matched-paired test)

To characterize the potential of CD34^+^ betamethasone-exposed cells in spleen and bone marrow repopulation, immune cell subsets were analyzed in these two lymphoid organs 20 weeks after transplantation. Figure 6 shows total cell numbers of splenic monocytes, cDC2s, pDCs, B lymphocytes, T lymphocytes (both CD4^+^ and CD8^+^), and CD4^+^ Treg cells. All subsets showed a trend to increase in the betamethasone group in comparison to the control group. Although non-significant, these results reflect a biological effect. In the betamethasone group, B cells were the most abundant (6.900.000, median of five mice), followed by T lymphocytes (308.000), pDCs (69.000), cDC2s (21.000), and monocytes (13.000), which correlates well to the human immune cell subsets found in peripheral blood during the follow-up. Figure 7 shows the total cell numbers of human cell subsets in the bone marrow. Similar to the spleen, all subsets displayed higher counts in the betamethasone group than in the PBS group. In the betamethasone group, B cells were the most abundant (850.000, median of five mice), followed by pDCs (200.000), monocytes (80.000), cDC2s (70.000), and T lymphocytes (15.000). In summary, a clear shift in the repopulation density was observed both in lymphoid and myeloid populations due to the effect of betamethasone at the end of the study.

## Discussion

In this work, we demonstrate for the first time that betamethasone modifies human cord blood CD34^+^ HSC phenotype enhancing engraftment ability. Phenotypic changes induced by the short exposure of HSC to betamethasone would promote the migration to bone marrow, thus improving engraftment in a competitive environment (suboptimal irradiation). Moreover, these CD34^+^ cells treated with betamethasone can differentiate in all the immune system cell lineages.

Betamethasone was selected as pre-treatment for CD34^+^ experimental transplantation for two reasons: (1) it is a commercially available and safe synthetic glucocorticoid used in the late phase of pregnancy to prevent respiratory distress syndrome in pre-term newborns (Sweet et al. 2019), and (2) glucocorticoids are involved in the ontogeny of the fetal immune system and other organs. Therefore, the fine-tuning of glucocorticoids is crucial since alterations in their concentration, for example, due to exogenous administration or aberrant endogenous production, can hugely impact fetal development (Solano et al. 2016). Moreover, these effects are maintained after birth due to epigenetic modifications (Bose et al. 2015; Zannas and Chrousos 2017).

We have previously described prenatal betamethasone’s effect on autoimmune diseases. In experimental T1D, an autoimmune disease caused by the destruction of insulin-producing β cells by T lymphocytes, we found a preventive effect of this drug. The mechanism of action was mainly through long-lasting alterations on the T cell receptor repertoire, thus protecting against β cell autoimmunity (Gieras et al. 2017; Perna-Barrull et al. 2019). These results point to a direct effect of betamethasone on HSC that will be maintained later in life. In humans, a preliminary study in a Spanish cohort shows that the developing immune system plasticity can be influenced by prenatal betamethasone, which could contribute to preventing autoimmune diabetes in subjects at risk (Perna-Barrull et al. 2022). Based on these data, we hypothesized that betamethasone could also exert beneficial effects on HSC to improve engraftment.

To assess that, we first determined the effect of betamethasone in cord blood HSC. We found that betamethasone alters CD34^+^ cell phenotype, mainly by increasing CXCR4 and reducing CD54 and HLA class II membrane expression. The increase of CXCR4 expression in CD34^+^ cells is a biomarker of enhanced migration potential to the bone marrow and spleen. It has been previously demonstrated that different glucocorticoids increase CXCR4 expression (Guo et al. 2017; Kahn et al. 2004; Kollet et al. 2001), but it has not been reported using betamethasone. Also, impairing the recognition of transplanted cells by the immune system of the host by reducing HLA class II molecules and CD54 will contribute to avoiding alloreactivity, reducing graft rejection, and increasing graft survival (Dustin 2014; Koga et al. 2020). These changes in the phenotype should contribute to the improved engraftment of betamethasone-exposed cord blood CD34^+^ cells in suboptimal conditions.

Then, we determined the effect of betamethasone on HSC engraftment. By irradiating mice with 2 Gy, we observed similar engraftment in mice transplanted with betamethasone-exposed or unexposed CD34^+^ cells, comparable to reported studies (Casamayor-Genescà et al. 2017). At the end of the follow-up, fluticasone and betamethasone show analogous engraftment, fitting well with similar changes in the phenotype observed in vitro, and despite a described higher affinity of fluticasone for the glucocorticoid receptor when compared to betamethasone (Daley-Yates 2015). It would be interesting to determine the differences induced in the gene expression profile by these two glucocorticoids and to correlate those results with their engraftment ability.

However, in a more graft-restrictive environment due to low irradiation (1 Gy), betamethasone-exposed CD34^+^ cells tended to improve engraftment results when compared to non-treated cells. The phenotype of betamethasone-exposed HSC points to this functional improvement, mediated, at least in part, by CXCR4 overexpression. This could contribute to a quick repopulation in the bone marrow niche before the host cells recover from the low irradiation, a condition that could be optimal for the host in a clinical setting. This fact can explain the differences observed in the total number of graft cells expanded between groups.

The percentages of the different subsets from 8 weeks after transplantation and until the end of the follow-up were similar to those previously reported (Audigé et al. 2017; Haworth et al. 2017). However, the use of adult NSG mice allowed us to perform an exhaustive follow-up of the immune system cells differentiated just after transplantation. Indeed, this is the first report on the evolution of the engraftment of CD34^+^ HSC from cord blood during the first 4 weeks after transplantation. Myeloid cells (dendritic cells and monocytes) compose the first wave of human cells in mouse peripheral blood. In human HSCT, a similar reconstitution has been described, being donor dendritic cells and monocytes detected 2–4 weeks after transplantation (Auffermann-Gretzinger et al. 2002; Turcotte et al. 2016). It would be interesting to analyze the ability of these myeloid cells to colonize the different secondary lymphoid organs and, at the same time, to determine their role in the establishment of the germinal centers. Because fetal cDC2s are immunosuppressive, but in adults, these cells induce T cell responses (McGovern et al. 2017), betamethasone could maintain this fetal tolerogenic action. We also observed higher engraftment in the spleen and bone marrow than in peripheral blood, as reported (Beyer and Muench 2017). This fact could be due, at least in part, to immunological differences in terms of chemokines that will hamper the mobilization of immune cells.

Interestingly, our results show a trend to increase Treg cells in the betamethasone-exposed CD34^+^ HSC, fitting well with previous results (Chen et al. 2006). It is well known that Treg cells are crucial for the immunosuppressive effects of glucocorticoids (Kim et al. 2020). At the same time, glucocorticoids promote the expression of FOXP3 in T cells, which induces Treg cell differentiation and function (Karagiannidis et al. 2004; Ugor et al. 2018). Thus, a Treg increase could be crucial to avoid rejection and graft versus host disease. Since betamethasone is prenatally administered and that fact correlates with protection against autoimmunity (Gieras et al. 2017; Perna-Barrull et al. 2022), it is reasonable to speculate that Tregs will play a role in homeostasis. This fact suggests that betamethasone-induced changes in HSC will be long-term maintained. Although glucocorticoid-driven epigenetic changes have been described, the specific effects of betamethasone on HSC have not been fully characterized. Morante-Palacios et al. (2022) reported the epigenetic mechanism of glucocorticoids that induce the differentiation from monocytes to tolerogenic dendritic cells. Understanding the role of this molecule in the development of immune system cells could help in the evolution of future therapeutic strategies.

We are well aware of the limitations of the study. First, this is a preliminary study, and the number of transplanted mice is scarce. Also, functional studies of the cell subsets differentiated in vivo would help to understand the descriptive results of longstanding betamethasone effects. Another limitation was that the UCB units used in this study were the ones discarded for clinical use and this is not the optimal substrate for the study and a clinical scale-up. Further validation in this setting will be required for clinical translation. Despite these limitations, these novel results show a clear benefit of using betamethasone in HSC transplantation. An important clinical implication would be that discarded UCB units or those banked with low CD34^+^ cell numbers could be recovered with a short-term treatment using betamethasone, as a pre-conditioning strategy.

In conclusion, betamethasone induces alterations in CD34^+^ HSC that improve engraftment in competitive conditions, leading to a faster recovery of the immune system and promoting the Treg lymphocyte subset that in turn will contribute to the survival of the engrafted cells.

## Data Availability

The datasets generated and/or analyzed during the current study are available from the corresponding author upon reasonable request.

## Declarations

### ARRIVE guidelines

We confirm that the manuscript follows the recommendations in the Arrive guidelines.
